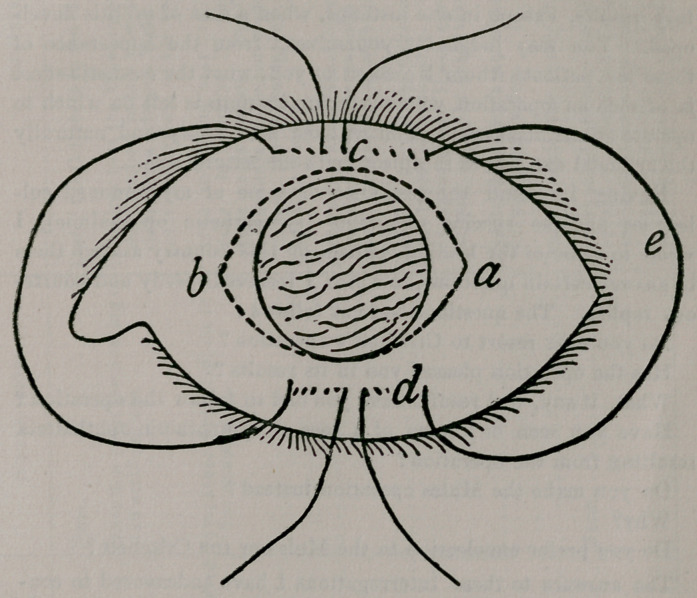# Corneal Abscission—A Report of Forty-one Operations

**Published:** 1906-01

**Authors:** J. M. Crawford

**Affiliations:** Atlanta, Ga.; Ophthalmic Surgeon Presbyterian Hospital, Tabernacle Infirmary, and Hospital for Incurables


### CORNEAL ABSCISSION—A REPORT OF FORTY-ONE OPERATIONS.*

By J. M. CRAWFORD, M.D., Atlanta.

Ophthalmic Surgeon Presbyterian Hospital, Tabernacle Infirmary, and Hospital for Incurables.

The object of this paper is to bring before you a subject with which the majority of you are more or less familiar, in print at least. I do not promise to throw any new light on the subject, but simply to give my personal experience, with the hope thereof of influencing some among you to lay aside prejudice and give to your patients the great benefit to be derived from this old and simple operation.

I say "old operation," since we find it spoken of more than one hundred and fifty years ago. Its revival was accomplished some thirty-five years ago by Critchett, of London, and later was modified by Knapp, of New York.

---

*Read before the Medical Association of Georgia, 1905.

### OPERATIVE PROCEDURE.

The operation, as you are aware, consists of the removal of the front portion of the ball together with the ciliary bodies and crystalline lens. The patient should be given chloroform or ether, as a local anesthetic is not sufficient. Personally, as I have stated on former occasions, I prefer the administration of chloroform because the psychic shock is much less and the after-effects better.

The primary operation of this kind was made by simply cutting off the anterior portion of the ball, leaving a stump formed by the sclera with the muscles attached. No stitches were taken to prevent the escape of the vitreous humor, and, strange though it may seem, this method was found to be better, in many instances, than enucleation.

### CRITCHETT'S OPERATION.

George Crittchett later improved on this method by inserting three or four curved needles threaded with silk through the ball from above downward, a little behind the ciliary bodies, after which he cut off the anterior portion of the ball in front of his needles, leaving a small margin of scleral through which he drew his needles. The scleral edges were brought together by means of these silk sutures, which were tied. The sutures were allowed to remain for six or eight days and were then removed.

### KNAPP'S OPERATION.

Knapp modified this operation by eliminating the curved needles and thread, closing the wound by external sutures, thus changing a very tedious operation into a simple one.

With the help of this cut I will endeavor to point out to you the successive steps in the operative procedure as I have practiced it. From the upper end of the vertical meridian (c), about four millimeters temporally, a curved needle is passed through the conjunctiva and the outer layers of the sclerotic, drawn out and introduced below the cornea at this point (d), passing through the conjunctiva and outer layers of the sclerotic in the same way as above the cornea, only in the opposite direction—nasally instead of temporally—and reappearing at the lower portion of the vertical meridian. The needle is removed and you have a loop (e) and the two ends of the thread resting on the temple. This process is repeated on the nasal side. The sutures being placed, you proceed to cut away that portion of the ball lying in front of them (a b), care being taken not to cut the threads and thereby delay the operation. The first step after the abscission is to let the ends out and tie the sutures. The ends of the temporal thread are drawn toward the nose, making of the loop a straight line and thus drawing the upper and lower edges of the temporal side of the wound together. The process is repeated on the nasal side, and when the sutures are tied, the wound has a puckered appearance similar to a pouch. The eye is then bandaged and the patient kept in bed until the wound heals, which it does speedily.

EVIDENCE.

As I have intimated in the beginning of this article, there is some prejudice in regard to this operation. The objection has been urged that the operation is frequently followed by sympathetic ophthalmia. I will say just here that I have within the last ten years made forty-one of these operations with entirely satisfactory results, except in one instance, when a case of cyclitis developed. You may judge for yourselves, from the appearance of these few patients whom I present to you, what the cosmetic effect is of such an operation where a natural stump is left on which to operate an artificial eye. You will see how easily and naturally the artificial eye moves in unison with the natural eye.

Having in mind the prejudice of some of my hononed colleagues and the specific objection (sympathetic ophthalmia), I wrote to some of the leading oculists of this country asking them to answer certain questions, to which I received speedy and courteous replies. The questions were as follows :

Do you ever resort to Critchett's operation?

Has the operation pleased you in its results?

What, if any, bad results have you had to follow the operation?

Have you seen or known of a case of sympathetic ophthalmia resulting from the operation?

Do you make the Mules operation instead?

Why?

Do you prefer enucleation to the Mules or the Critchett?

The answers to these interrogations I have endeavored to condense into tabular form, as follows:

In answer to my letter, G. E. de Schweinitz, of Philadelphia, kindly sent me a copy of his paper which he read at the Thirteenth International Congress of Medicine at Paris in 1900.

In this paper twenty-eight operators give evidence in regard to abscission, sixteen of them recording one hundred and eighty-six cases, while twelve do not state the number of their operations.

Out of the mass of evidence Dr. de Schweinitz deduces the following: "Sympathetic inflammation after abscission must be uncommon, although from the character of the operation, it might be expected. The English committee reports only three cases, and I have secured only one positive expression of opinion upon this complication, and that is from Dr. Richard Derby, of New York, who says: 'My experience has led me to regard the operation of abscission as an unsafe surgical procedure, and one that both in my hands and in the hands of other surgeons has been followed by sympathetic ophthalmitis.' No particulars are given."

Out of twenty-eight operators and one hundred and eighty-six operations of abscission, one operator seriously objects to the procedure, and the English committee reports three cases of sympathetic ophthalmia. Not a very large percentage I take it.

From the following table, which I take the liberty to quote from de Schweinitz's paper, you will readily see that my position of favoring abscission is not an isolated one, but that the position taken by me and the good results are alike shown by many.

WHEN INDICATED.

Of course the operation must be limited to certain diseased conditions of the eye, and is especially indicated in cases of staphyloma of the cornea.

Eyes in which suppuration has begun may be abscised without danger, unless the process has involved the surrounding orbital tissues, or already begun to extend posteriorly so that an aseptic socket would be difficult to obtain.

Staphylomatous eyeballs in children, when not inflamed, may be treated by the operation of abscission or keratectomy, though it is possible in after-years for the stump to undergo calcareous or osseous change, which may cause sympathetic irritation in the other eye and require enucleation. Such cases I have never seen.

CONTRAINDICATED.

Eyes so diseased or injured that they have already excited sympathetic ophthalmitis, or eyes containing malignant growths, should be enucleated. I do not favor evisceration, having made the operation but a few times.

Shrunken eyes (excessive phthisis bulbi) should be enucleated. When a patient is very old or feeble, enucleation is preferable to abscission.

I take it, however, that the field for the operation has been too limited. This operation can often be made to a great advantage to the life of a patient where it is made for the cosmetic effect. Take the case, for instance, of a young business man or a young girl growing up with a blind eye, with a white cornea that attracts attention. In these instances you afford a great deal of comfort to your patients by making this operation and inserting an artificial eye that moves with its fellow eye, and at the same time you do not, as many have thought, endanger the good eye with sympathetic ophthalmia. I have never made Mules' operation, since it did not appeal to me. I had rather have a stump made from the natural vitreous humor than one made by the introduction of a glass or silver ball, since these balls often come out, and also since we have cases on record where sympathetic ophthalmia has been caused by this (Mules') operation.

## Figures and Tables

**Figure f1:**